# A 14-Year-Old Boy with Unusual Presentation of Respiratory Distress

**DOI:** 10.1155/2016/7313942

**Published:** 2016-12-01

**Authors:** Adam W. Powell, Samuel Hanke, James S. Tweddell, Nicolas Madsen

**Affiliations:** ^1^Division of Pediatric Cardiology, Cincinnati Children's Hospital, Cincinnati, OH, USA; ^2^Division of Pediatric Cardiovascular Surgery, Cincinnati Children's Hospital, Cincinnati, OH, USA

## Abstract

There are multiple cardiac etiologies for wheezing and respiratory distress which require a high degree of suspicion for the pediatrician to diagnose. We present a case of a patient with a history of long-standing mild persistent asthma with minimal improvement on controller and bronchodilator therapies who presented to the emergency room with acute respiratory distress. When he demonstrated a lack of improvement with traditional respiratory therapies, additional etiologies of respiratory distress were considered. Ultimately an echocardiogram was performed, which revealed the diagnosis of cor triatriatum. He underwent surgical resection of his accessory membrane and has had no additional symptoms of asthma since repair.

## 1. Introduction

Cor triatriatum is a rare congenital heart defect that can present with isolated respiratory distress mimicking other pulmonary conditions. While this is an uncommon diagnosis for the pediatrician to make in the patient with respiratory distress, it is a part of a larger differential diagnosis of cardiac lesions that may present with respiratory distress or recurrent, treatment refractory wheezing. For cor triatriatum, a high index of suspicion is required to make the appropriate diagnosis in a patient with respiratory symptoms that do not respond as expected to usual interventions. The diagnosis is best made by echocardiogram. The treatment of choice is surgical resection of the accessory membrane that is generally well tolerated. Long-term follow-up with pediatric cardiology is required after resection to monitor for rare sequelae.

## 2. Case Presentation

A 14-year-old boy presented to the hospital with respiratory distress for six hours. His past medical history was remarkable for long-standing mild persistent asthma with reports of only intermittent improvement in symptoms with Albuterol. On the day prior to this visit, he reported new fatigue and myalgia following a band practice. Later in that evening he developed emesis, cough, congestion, difficulty breathing, and a temperature of 104°F. Albuterol inhaler was administered twice prior to arrival in the hospital without improvement in symptoms. Upon arrival to the emergency room, his oxygen saturations were 70–80% in room air. On physical exam, he was in mild respiratory distress with diminished breath sounds bilaterally, without wheezing. The remainder of his physical exam was unremarkable.

**Figure 1:**
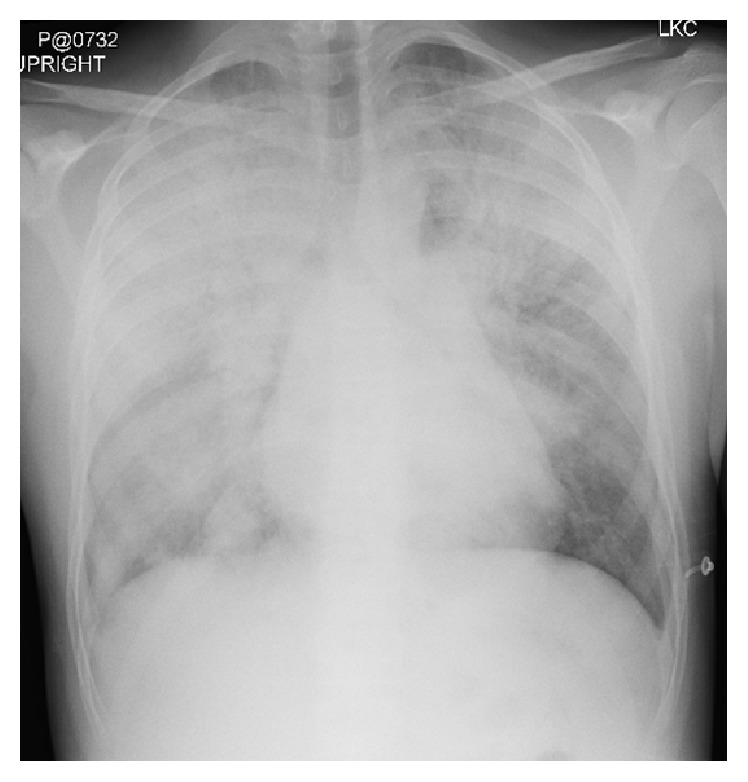
Chest X-ray at presentation demonstrating extensive airspace disease that was interpreted as multifocal pneumonia versus pulmonary edema.

Initially asthma exacerbation was suspected as the etiology of his respiratory distress and he was given an Albuterol-Ipratropium nebulized treatment and IV Methylprednisolone. The patient's lack of response to these therapies caused the Emergency Department team to expand the differential diagnosis beyond asthma. A racemic epinephrine treatment was given without improvement. Laboratory studies were remarkable for initial venous blood gas with pH of 7.25 and PCO2 50 mmHg and a white blood cell count of 24,000 K/mcL with a left shift. The serum renal panel and liver function test were unremarkable. Blood cultures and respiratory viral testing were obtained. His initial chest X-ray revealed extensive airspace disease bilaterally consistent with multifocal pneumonia versus pulmonary edema (Figure 1). His respiratory support was escalated and he was transferred to the pediatric intensive care unit (PICU).

Upon arrival in the PICU, his condition worsened with an inability to maintain normal oxygen saturations on BiPAP with 100% FiO2. He was intubated given his deteriorating clinical status. Intubation and ventilation were notable for immediate frothy drainage from the endotracheal tube and the need for high ventilator pressures consistent with diffuse pulmonary edema. Additionally, he was started on an epinephrine drip due to hypotension refractory to two liters of normal saline. He was started on Vancomycin, Ceftriaxone, and Tamiflu while awaiting culture results. Ultimately, an echocardiogram was obtained due to the degree of pulmonary edema and his respiratory deterioration despite multiple ventilation strategies.

**Figure 2:**
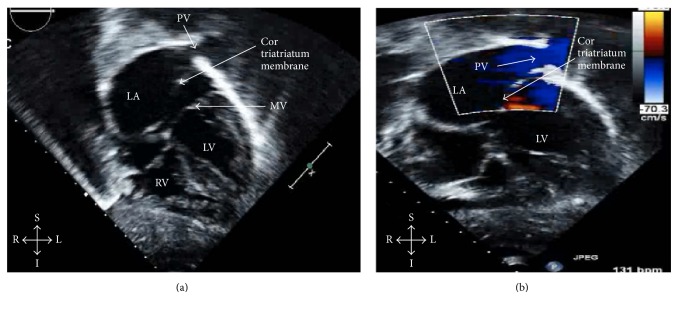
Subcostal coronal view on echocardiogram (a) demonstrates cor triatriatum with a membrane separating the pulmonary veins from the left ventricle inflow and (b) is a Doppler image demonstrating restricted flow through the opening of the cor triatriatum membrane with high-velocity flow through a dilated left pulmonary vein. LV: left ventricle, RV: right ventricle, LA: left atrium, RA: right atrium, MV: mitral valve, PV: pulmonary vein, S: superior, I: inferior, R: right, and L: left.

**Figure 3:**
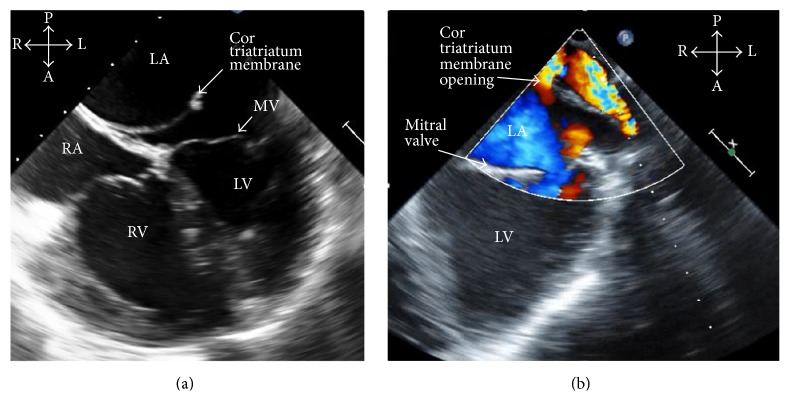
Transesophageal echocardiogram (a) is a midesophageal four-chamber view demonstrating cor triatriatum with a membrane separating the pulmonary veins from the left ventricle inflow and (b) is a midesophageal two-chamber view with Doppler flow demonstrating restrictive flow through the opening of the cor triatriatum membrane. LV: left ventricle, RV: right ventricle, LA: left atrium, RA: right atrium, MV: mitral valve, S: superior, I; inferior, R: right, L: left, A: anterior, and P: posterior.

The initial transthoracic echocardiogram was suggestive of cor triatriatum with a membrane separating the left atrium into two chambers (Figure 2(a)) and Doppler flow demonstrating the pulmonary veins enter the left atrium on the proximal side of the atrial membrane (Figure 2(b)). Additionally, the echocardiogram demonstrated right ventricular hypertension (pressure estimated to be greater than half the systemic blood pressure). No other congenital heart defects were noted. A transesophageal echocardiogram confirmed the diagnosis of severely restrictive cor triatriatum with a mean gradient of 27 mmHg across the membrane (Figure 3). As a result of this diagnosis, the patient was brought to the operating room for emergent resection of his cor triatriatum membrane.

Following his repair, he remained intubated with vasopressor support for two days. His immediate postoperative chest X-ray demonstrated improvement in his pulmonary edema suggesting that a primary respiratory etiology was not the principal factor for his presentation. His chest X-ray improved further over the course of his hospitalization with diuresis. His initial blood cultures were negative and the respiratory viral panel was positive for influenza for which he completed a 5-day course of Tamiflu. He was discharged on furosemide twice daily. During his cardiology ambulatory clinic visits, his chest X-ray normalized and his diuretics were discontinued. Once postoperative activity restrictions were lifted, he was able to participate in a vigorous mountain hike without the need for bronchodilators, something that he was unable to do prior to surgery. He continues to have long-term follow-up with cardiology to monitor for left atrium tissue overgrowth and pulmonary vein stenosis.

## 3. Discussion

Wheezing is a common symptom among pediatric patients with around 50% of children reporting at least a single episode of wheezing by five years of age [1]. While asthma is the most common reason for recurrent wheezing, there is a large differential diagnosis. Cardiac anomalies represent a less frequent cause of wheezing and therefore require a high index of suspicion. The two most common mechanisms of cardiac induced wheezing are direct obstruction of the bronchus (e.g., vascular rings and slings) and conditions that cause elevated pulmonary venous pressure (e.g., pulmonary venous obstructive lesions, mitral stenosis, and cardiomyopathy) (Table 1) [2]. Of note, tracheobronchial obstructive lesions can present with either stridor or wheezing while conditions that cause elevated pulmonary venous pressure do not typically present with stridor [2, 3].

**Figure 4:**
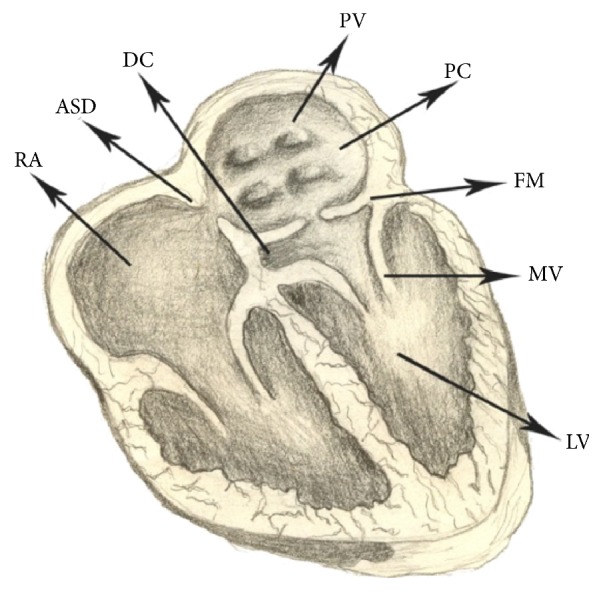
The classic form of cor triatriatum, demonstrating a fibromuscular membrane in the left atrium separating the proximal chamber from the distal chamber containing the mitral valve and left ventricular inflow. Of note, this drawing contains an atrial septal defect which is not required to be present for the diagnosis of cor triatriatum. LV: left ventricle, RV: right ventricle, LA: left atrium, RA: right atrium, MV: mitral valve, ASD: atrial septal defect, PV: pulmonary vein, PC: proximal chamber, and FM: fibromuscular membrane. (J Tehran Heart Cent. 2010 Summer; 5(3): 153–155. Published online 2010 Aug 31. © Copyright Policy: open-access, License.)

Cor triatriatum is a rare congenital heart disease representing <0.1% of all congenital cardiac malformations. It was first reported on by Church in 1868 as a triatrial heart [4]. It is characterized by a fibromuscular membrane which separates the left atrium into a proximal segment that receives the pulmonary veins and a distal chamber that contains the mitral valve (Figure 4). Cor triatriatum may very rarely occur on the right side of the heart (cor triatriatum dexter) with around 300 cases reported in the literature [5]. The pathophysiology of these lesions differs greater with cor triatriatum dexter being related to excessive eustachian valve tissue [6]. Cor triatriatum is associated with other congenital heart defects 80% of the time, most commonly atrial septal defects, and partial anomalous pulmonary venous return [7].

Symptoms occur when there is restriction of flow through the fibromuscular membrane and vary based on the degree of obstruction. This mechanical obstruction causes slowing of pulmonary venous flow and results in pulmonary hypertension. Patients who have no obstruction through the fibromuscular membrane are often asymptomatic and are diagnosed incidentally later in life. Patients with a severely restrictive membrane can present as early as in the neonatal period with respiratory distress [8]. Common presenting symptoms include recurrent respiratory infections, tachypnea, and failure to thrive [9]. More rarely, as was the case in the 14-year-old boy described above, patients present with isolated wheezing as the sole symptom for cor triatriatum [10]. Typically when older children and adults present with symptoms, it is secondary to fibrosis and calcification of the membrane resulting in progressive obstruction [11].

Clinical exam findings are variable, but auscultation may include a diastolic murmur or a prominent P2 component of the second heart sound if pulmonary hypertension is present [9]. In cases when the membrane is not restrictive, the physical exam is often normal [8]. Electrocardiogram is usually normal unless there is right ventricular hypertrophy and right axis deviation as a result of pulmonary hypertension [12]. Chest X-ray may demonstrate pulmonary vascular congestion and pulmonary edema [12]. Ultimately, echocardiogram provides the greatest diagnostic utility. It is a sensitive and specific test to identify the presence of the thin fibromuscular membrane bisecting the left atria (Figure 2) [7]. Doppler flow through the membrane is used to determine the degree of restriction (Figure 3) [9].

Treatment strategies are dependent on the degree of symptoms. An echocardiographic finding of a nonrestrictive septum can be followed clinically for the development of pulmonary hypertension without treatment. If a patient presents with signs of pulmonary venous obstruction, medical management with diuretics and preload reduction can be attempted prior to the definitive treatment of surgical correction. Surgery involves resection of the accessory atrial membrane and it is generally well tolerated with over 90% of patients remaining symptom-free five years after repair [13]. Patients will require long-term serial echocardiograms to assess for progressive pulmonary vein stenosis and left atrial tissue overgrowth [14].

## Figures and Tables

**Table 1:** Examples of cardiac causes of wheezing and respiratory distress.

Bronchial obstructive	Pulmonary venous obstructive	Pulmonary vascular congestion
Double aortic arch	Pulmonary vein stenosis	Ventricular septal defect
Right aortic arch with aberrant left subclavian and left ductus arteriosum	Anomalous pulmonary venous return	Patent ductus arteriosus
Pulmonary artery sling	Mitral stenosis	Cardiomyopathy
	Cor triatriatum	Anomalous left coronary artery from the pulmonary artery
